# LFC-YOLO: A Lightweight Feature-Complementary YOLO Framework for Small Object Detection in UAV-Based Visual Sensing

**DOI:** 10.3390/s26165037

**Published:** 2026-08-08

**Authors:** Bin Chen, Qiang Fan, Xiaoxiong Zhang, Zhenrong Zhang, Jiancheng Sun, Zhihui Ge, Laiyuan Tong, Xuebin Tang

**Affiliations:** 1 School of Computer, Electronics and Information, Guangxi University, Nanning 530004, China; 2 The Sixty-Third Research Institute, National University of Defense Technology, Nanjing 210023, China; 3 National Key Laboratory of Big Data and Decision, National University of Defense Technology, Changsha 410073, China; 4 Guangxi Key Laboratory of Multimedia Communications and Network Technology, Guangxi University, Nanning 530004, China

**Keywords:** UAV imagery, small object detection, lightweight object detection, high-resolution feature pyramid, feature fusion, YOLOv8

## Abstract

Object detection in unmanned aerial vehicle (UAV)-based visual sensing is important for aerial monitoring and intelligent perception. However, it remains difficult because camera-captured aerial images often contain small targets, cluttered backgrounds, occlusion, and limited edge-computing resources. We propose LFC-YOLO, a lightweight feature-complementary detector for small objects in UAV imagery. The main component of LFC-YOLO is the Tiny Object-Specific Detection Architecture (TSD-Arch), which removes redundant computation from deep layers and builds a shallow high-resolution feature pyramid to preserve localization cues for small targets. To reduce the extra cost introduced by high-resolution feature fusion, lightweight GSConv is integrated into the reconstructed neck. In addition, we embed a Feature Complementary Mapping (FCM) block into the C2f backbone structure and form a C2f-based Feature Complementary Mapping (C2f-FCM) module. This module combines semantic and spatial information and reduces interference from complex backgrounds. Experiments on VisDrone2019 show that LFC-YOLO improves the mean average precision at an intersection-over-union threshold of 0.5 (${\mathrm{mAP}}_{50}$) by 4.0 percentage points over YOLOv8s while reducing the number of model parameters by 73.9%. Additional evaluation on UAVDT shows that the proposed design remains effective across different UAV scenarios.

## 1. Introduction

**Figure 1:**
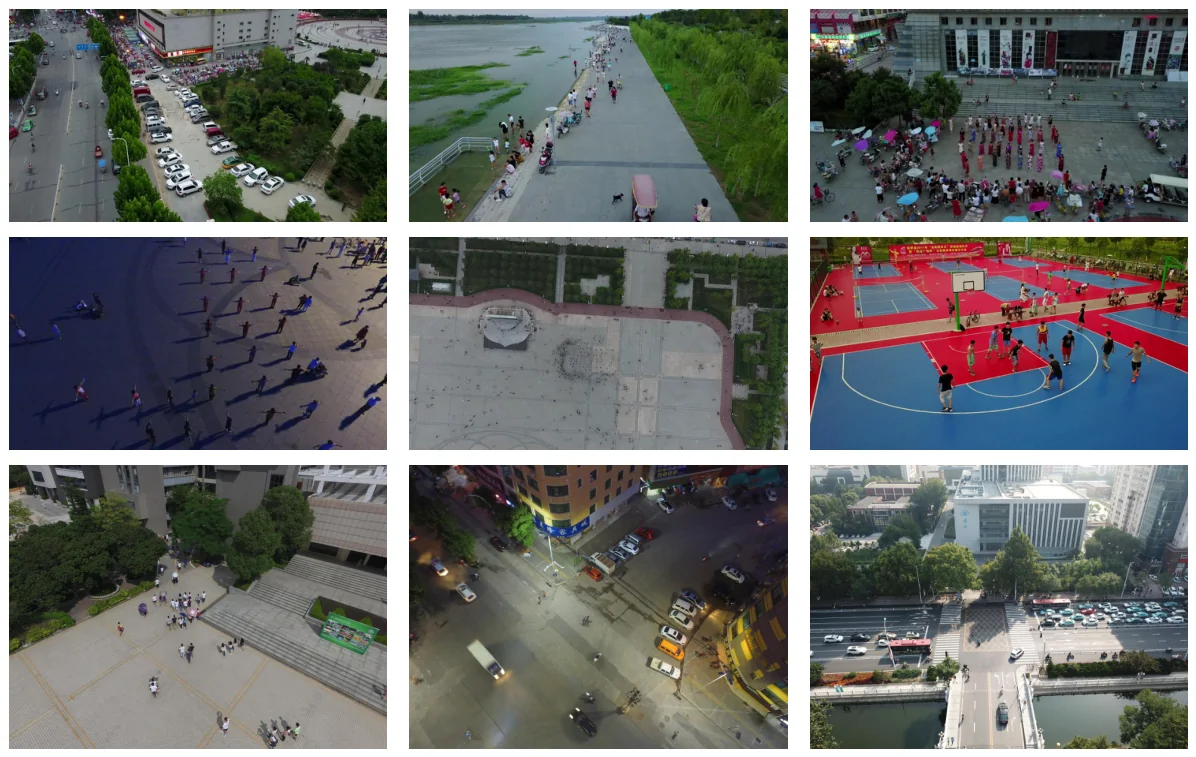
Examples of common difficulties in UAV aerial imagery, including background interference, large-scale variation, and dense small-object distributions.

Unmanned aerial vehicles (UAVs) are now widely used in smart city management, agricultural monitoring, disaster response, and rescue operations because they are easy to deploy, inexpensive to operate, and able to capture wide-area views [1]. Compared with natural scene images, however, UAV aerial images are more difficult for object detectors. They often contain oblique viewing directions, large changes in flight altitude, and broad scene coverage, all of which weaken the reliability of conventional detection methods. Designing robust detectors for UAV platforms is further limited by two practical issues. Many targets in aerial images are small and occupy only a few pixels, so their spatial features can be severely weakened or nearly lost after repeated downsampling in deep networks. As shown in Figure 1, UAV images also contain complex backgrounds, strong illumination changes, and frequent occlusions, which often cause missed detections and false positives [2]. These difficulties are compounded by the limited computation and storage available on edge devices, including UAV platforms, where detectors must remain lightweight while supporting efficient inference under limited resources.

Several lines of work have been proposed for these problems [3]. Liu et al. [4] and Tian et al. [5] improve small-object feature extraction using dual-network verification and optimized network structures. To reduce the effect of complex backgrounds, Li et al. [6] and Yang et al. [7] use semantic guidance and attention mechanisms to focus detection on object-dense regions and obtain more accurate boundaries. Recent Transformer-based detectors [8,9] model global context and frequency-domain information to reduce feature degradation. These methods can improve accuracy, but multi-branch structures and large computational workloads may increase inference latency [10]. Lightweight detectors, such as the method of Shao et al. [11], reduce parameter count with compact architectures; however, excessive compression can also remove useful representation capacity and lower detection accuracy [12].

Reducing parameter count is important not only for model storage and deployment but also for controlling unnecessary model capacity. Statistical learning theory suggests that model capacity should be considered together with the amount of available training data [13,14].

Given the strong baseline performance of YOLOv8s [15], we use it as the base detector and adapt its feature hierarchy to UAV aerial imagery. LFC-YOLO contains two main components: the Tiny Object-Specific Detection Architecture (TSD-Arch) and the C2f-based Feature Complementary Mapping (C2f-FCM) module. TSD-Arch adjusts the feature hierarchy for tiny-object detection by removing the conventional $P_{5}$ branch and building a shallow high-resolution feature pyramid, which improves localization for small targets. To keep the cost of high-resolution feature extraction under control, we add the lightweight GSConv module to the neck. C2f-FCM combines low-level spatial details with higher-level semantic information. Experiments on VisDrone2019, together with independent training and evaluation on UAVDT, show that LFC-YOLO achieves a practical balance between detection accuracy and model complexity.

## 2. Methods

### 2.1. Overall Architecture

**Figure 2:**
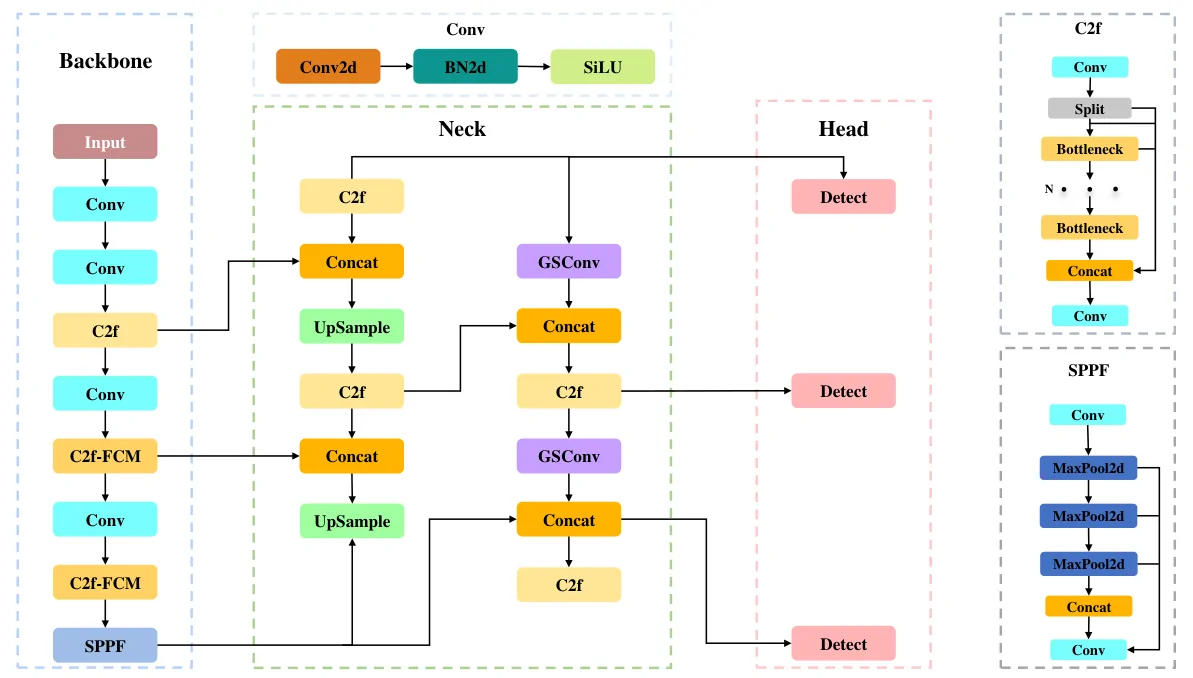
The structure of the LFC-YOLO network.

LFC-YOLO is built on YOLOv8s [16] to improve small-object detection in complex UAV scenes while keeping the model compact [17]. Its overall structure is shown in Figure 2. We first design the Tiny Object-Specific Detection Architecture (TSD-Arch) to preserve features for very small objects. TSD-Arch removes the conventional deep branch $P_{5}$ and rebuilds the cross-layer fusion paths in the neck network [18]. It also adds the high-resolution layer $P_{2}$ to the feature pyramid, which helps retain fine-grained localization cues. Since extracting high-resolution $P_{2}$ features increases computation, we replace standard convolutions in the reconstructed neck with the lightweight GSConv module [12]. We further insert the C2f-FCM module into the backbone network to combine shallow spatial details and deep semantic information through bidirectional feature complementation. The module uses a compact decoupled structure to reduce background interference while keeping the backbone parameter-efficient. Together, these changes reduce deep-stage capacity and concentrate representation on the spatial scales that are most relevant to dense and tiny objects.

### 2.2. TSD-Arch

Objects in UAV aerial imagery often occupy only a very small number of pixels [4]. The standard YOLOv8s model uses a deep feature pyramid $\left\{P_{3},P_{4},P_{5}\right\}$, with a maximum downsampling rate of 32×. After five strided convolutions, a tiny object smaller than 16×16 pixels is theoretically mapped to less than 0.5×0.5 pixels on the $P_{5}$ feature map [19]. At this scale, high-frequency spatial details of weak targets are easily lost. Deeply downsampled features provide coarse semantic context but contain less direct localization detail for targets occupying only a few input pixels.

Existing methods such as TPH-YOLOv5 [20] typically keep the $P_{5}$ layer and add high-resolution prediction heads or computation-heavy Transformer modules. These stacked designs can improve tiny-object recall, but they also increase the number of parameters and inference latency, which is unfavorable when model capacity and practical speed are constrained. To better match the network structure to the object scale, we propose TSD-Arch. Rather than adding local enhancement modules on top of the original network, TSD-Arch uses deep truncation and shallow reconstruction to improve tiny-object detection through a parameter-efficient design.

#### 2.2.1. Deep Truncation and Computational Trade-Off

At the macro-topological level, TSD-Arch retains the early C2f block and its following stride-2 convolution in YOLOv8s and truncates only the deepest $P_{5}$ stage, which has the largest channel dimension and the coarsest spatial resolution in the original pyramid. The maximum downsampling rate is therefore limited to 16×, corresponding to the $P_{4}$ layer. To retain part of the global receptive field after truncating the deep stage, we move the Spatial Pyramid Pooling-Fast (SPPF) module [15] to the end of the $P_{4}$ stage. Rather than adding further blocks to the original detector, we remove the $P_{5}$ stage and reorganize the remaining feature hierarchy. The complete TSD-Arch configuration reduces the parameter count by approximately 70.3% relative to YOLOv8s, lowering model storage and memory requirements. Its shallow high-resolution feature maps increase the nominal number of floating-point operations (FLOPs). The practical accuracy and latency effects of this architectural trade-off are evaluated using the unified end-to-end timing protocol in Section 3.

#### 2.2.2. Shallow High-Resolution Pyramid Construction

TSD-Arch further introduces a high-resolution $P_{2}$ stage into the Path Aggregation Network (PANet) [21], forming a shallow $\left\{P_{2},P_{3},P_{4}\right\}$ feature pyramid. This design reduces the loss of tiny-object features during forward propagation. In the top-down path, semantic information is passed from $P_{4}$ to $P_{2}$. In the bottom-up path, however, applying standard convolutions to large high-resolution feature maps would add considerable computation. To limit this cost in the neck network, we replace standard downsampling convolutions with the lightweight GSConv module [12] with a stride of 2. GSConv is used to reduce the computational cost of the high-resolution bottom-up path while maintaining sufficient feature representation for tiny-object localization. The final decoupled prediction heads operate directly on the three shallow high-resolution feature maps [20]. This structural reorganization improves tiny-object localization in UAV scenarios while keeping the model compact.

### 2.3. GSConv Module Integration

As discussed in Section 2.2, TSD-Arch rebuilds the neck network as a high-resolution $\left\{P_{2},P_{3},P_{4}\right\}$ pyramid to preserve fine-grained details for tiny objects. This design improves localization, but applying standard convolutions (SCs) to large feature maps brings a high computational cost. Depthwise separable convolution (DSC) [22] can reduce the number of parameters (Params) and floating-point operations (FLOPs), but it usually has weaker feature extraction ability than SC.

**Figure 3:**
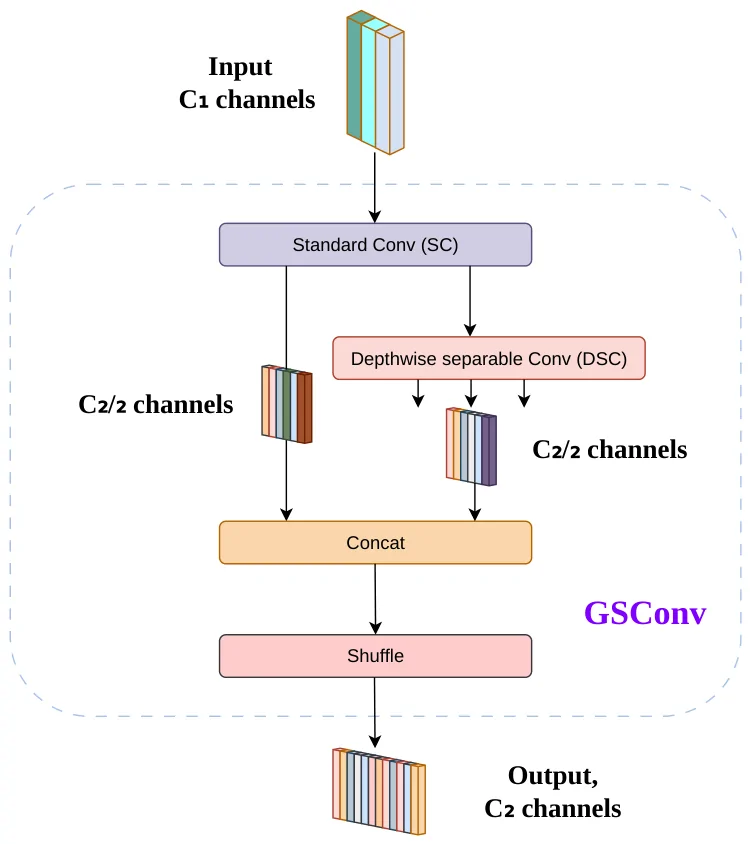
Detailed architecture of the integrated GSConv module.

To keep the reconstructed neck network efficient while preserving feature representation, we use the GSConv module [12]. GSConv follows a hybrid convolution design. As shown in Figure 3, it combines SC and DSC and then mixes their outputs through a Channel Shuffle operation [23].

Let the input and output channel numbers be $C_{1}$ and $C_{2}$, respectively. The input feature map is first passed through a standard convolution to extract dense channel information, producing an intermediate feature map with $C_{2}/2$ channels. This intermediate feature map is then fed into a depthwise separable convolution to extract spatial information, producing another $C_{2}/2$ channels. The two feature groups are concatenated along the channel dimension, after which channel shuffle is applied to encourage interaction between channel-wise and spatial features. In this way, GSConv retains much of the feature representation provided by standard convolution while reducing computational cost.

We apply GSConv only to the reconstructed neck network rather than to the whole backbone. Stacking GSConv modules in the early stages would deepen the network and increase memory access overhead, which may slow down inference. By contrast, the TSD-Arch neck processes feature maps that already contain multi-scale context and have large spatial sizes, where convolution is a major source of computation. Using GSConv at this stage reduces the computational cost while preserving sufficient feature representation for tiny objects in UAV imagery.

### 2.4. C2f-FCM Module

**Figure 4:**
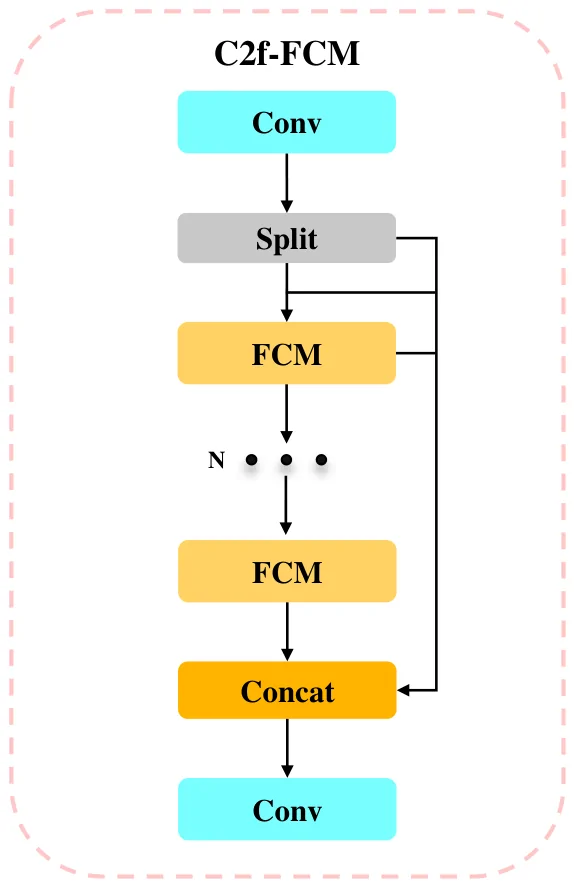
Structure of the C2f-FCM network.

**Figure 5:**
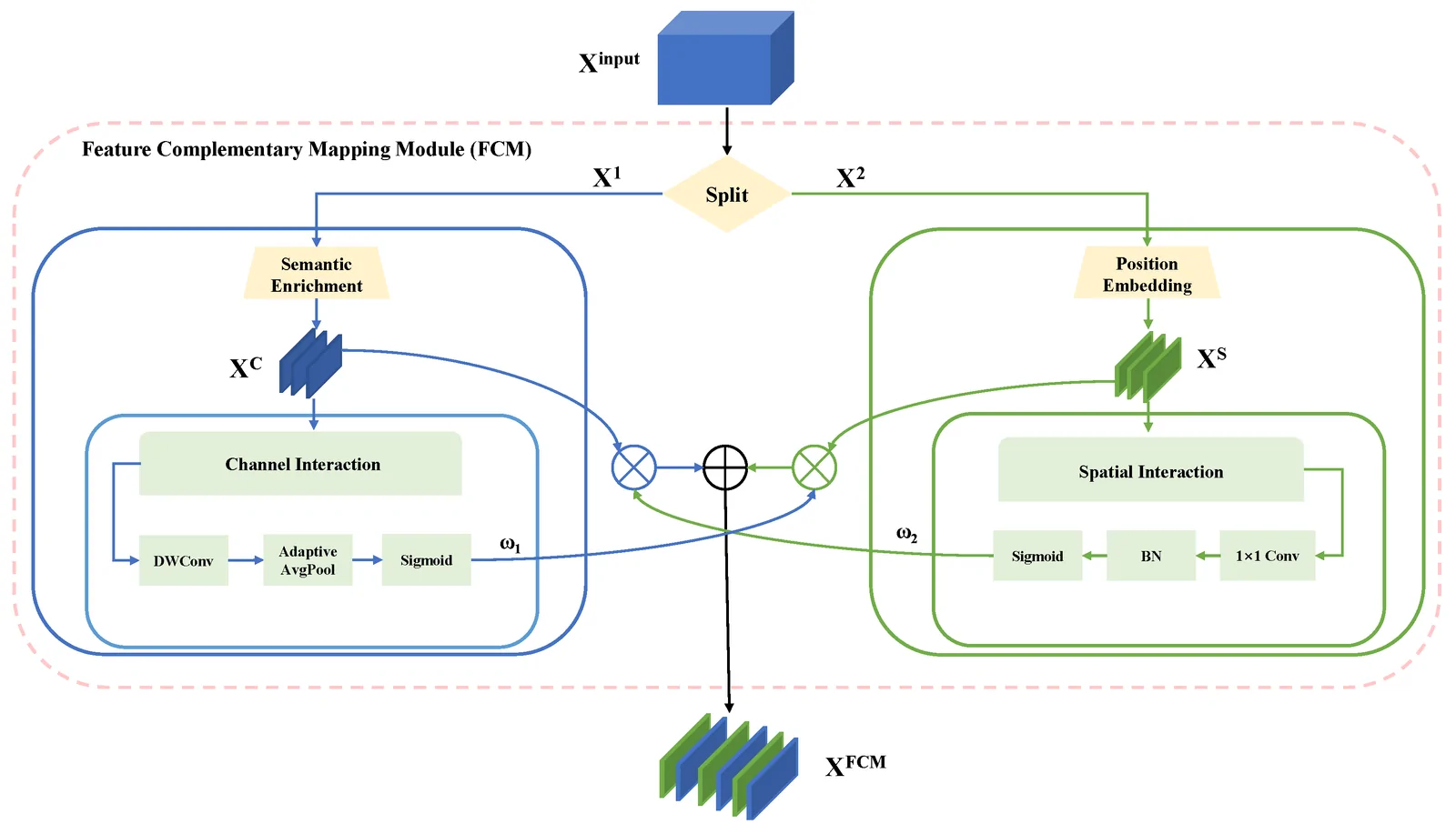
Structure of the FCM network.

The C2f module is the main feature extraction block in YOLOv8s [16]. It splits the input feature map along the channel dimension. One branch is sent directly to the final concatenation stage, while the other passes through several cascaded bottleneck layers. The outputs from these branches are then concatenated to strengthen feature representation. This structure is effective for semantic feature extraction, but repeated convolutions may weaken spatial coordinates and texture details for small objects in UAV images, where targets often occupy only a few pixels. Attention mechanisms are commonly used to reduce feature degradation in small-object detection, yet standard designs such as SE [24] and CBAM [25] are not always well suited to UAV imagery. SE uses global average pooling, which can discard spatial coordinates that are important for small targets. CBAM applies attention sequentially, which may smooth out shallow fine-grained features during forward propagation. To address these issues, we integrate the Feature Complementary Mapping (FCM) block into the C2f structure and form the C2f-FCM module, as illustrated in Figure 4 and Figure 5. The FCM block uses a decoupled design to extract high-dimensional semantic information ($X^{C}$) and shallow spatial information ($X^{S}$) in parallel, followed by cross-branch fusion. This design makes object-related features more salient while preserving the high-frequency details of small objects. The lightweight DWConv design also reduces convolutional cost within the module, supporting a more parameter-efficient backbone.

As shown in Figure 5, the FCM block [26] is used inside C2f-FCM to split, transform, complement, and aggregate features, thereby reducing the loss of spatial position information and narrowing the gap between semantic and spatial features for small objects. Given an input feature map $X^{\mathrm{input}}\in R^{C\times H\times W}$, the module is described by the following two stages.

#### 2.4.1. Decoupling and Transformation

The input feature map $X^{\mathrm{input}}\in R^{C\times H\times W}$ is first split along the channel dimension into two sub-features according to a ratio α: $X^{1}\in R^{\alpha C\times H\times W}$ and $X^{2}\in R^{(1-\alpha )C\times H\times W}$.

To obtain semantic and spatial representations with aligned tensor dimensions for later fusion, the two branches are processed by separate convolutional transformations:

(1) $$\left(X^{C},X^{S}\right)=\left({\mathrm{Conv}}_{3\times 3}\left(X^{1}\right),{\mathrm{Conv}}_{1\times 1}\left(X^{2}\right)\right),$$

where $X^{C},X^{S}\in R^{C\times H\times W}$. Here, $X^{C}$ captures high-dimensional channel semantics, while $X^{S}$ retains shallow spatial position information. In our implementation, α is set to 0.5, and both branch transformations output *C* channels to enable element-wise cross-branch weighting and aggregation.

#### 2.4.2. Bidirectional Weighting and Aggregation

Since the two branches extract semantic and spatial information separately, $X^{C}$ and $X^{S}$ may become misaligned. The module therefore exchanges information at both the channel and spatial levels. Depthwise convolution (DWConv) is first applied to $X^{C}$ to model spatial dependencies within each channel. The resulting feature is passed through adaptive global average pooling (Adaptive AvgPool) and a Sigmoid activation function to generate the channel attention weight $\omega_{1}\in R^{C\times 1\times 1}$. In parallel, $X^{S}$ is compressed along the channel dimension using a 1×1 pointwise convolution, batch normalization (BN), and a Sigmoid activation function, producing the spatial attention weight $\omega_{2}\in R^{1\times H\times W}$. These two attention weights are applied to the opposite branches, and the final feature $X^{FCM}$ is obtained by feature aggregation:

(2) $$X^{FCM}=\left(X^{C}⊗\omega_{2}\right)⊕\left(X^{S}⊗\omega_{1}\right),$$

where ⊗ denotes element-wise multiplication with broadcasting, and ⊕ denotes element-wise addition.

The C2f-FCM module retains the cross-stage gradient propagation of the original C2f module while using the FCM block to perform semantic–spatial feature complementation within this gradient-flow structure. Rather than directly inserting an isolated attention block, C2f-FCM embeds FCM into the C2f feature extraction framework and complements the shallow high-resolution detection hierarchy introduced by TSD-Arch. This design passes shallow spatial position information to deeper layers and strengthens feature representation for small-object detection under complex UAV backgrounds.

## 3. Experiments and Results

### 3.1. Implementation Details

We ran all experiments on Ubuntu 24.04.3 LTS. The workstation was equipped with an Intel Xeon Gold 6133 CPU at 2.50 GHz and a single NVIDIA GeForce RTX 4090 GPU with 24 GB of memory. The software stack used Python 3.8.20, PyTorch 2.1.0, and CUDA 12.1.

All models were initialized with pretrained weights and trained on the GPU. Input images were processed using the standard Ultralytics letterbox procedure and placed on a 640×640 canvas. This procedure preserves the original aspect ratio and pads the remaining area rather than independently stretching the horizontal and vertical dimensions. The same input setting was used for all models to ensure a consistent and fair comparison. We trained each model for up to 300 epochs with a batch size of 8. Early stopping with a patience of 100 epochs was used, so training could finish before 300 epochs when the validation performance no longer improved. Parameters were optimized with Stochastic Gradient Descent (SGD), using an initial learning rate of 0.01, a momentum of 0.937, and a weight decay of 0.0005. Mosaic augmentation (mosaic = 1.0) [27] was enabled during training, and Automatic Mixed Precision (AMP) [28] was used to improve training efficiency. For qualitative visualization, we used the default prediction settings of the Ultralytics YOLOv8 implementation. Quantitative mAP results were computed using the standard validation protocol rather than a single fixed visualization threshold.

To measure practical inference efficiency, we conducted a controlled timing evaluation of the end-to-end detection pipeline on the 548-image VisDrone2019 validation set using the same NVIDIA GeForce RTX 4090. All models were tested at an input size of 640×640, a batch size of 1, and FP32 precision. Each model was warmed up and then evaluated over the complete validation set in five repeated runs, with the model order varied across runs. The reported latency is the mean per-image detection-pipeline time, including preprocessing, network inference, and postprocessing but excluding image loading and external system overhead. The tests used a confidence threshold of 0.5, a non-maximum suppression IoU threshold of 0.45, and a maximum of 300 detections per image. FPS was calculated as 1000 divided by the mean latency in milliseconds. Table 1 reports the full set of training hyperparameters.

### 3.2. Dataset

In this study, we use two publicly available UAV object detection datasets: VisDrone2019 [29] and UAVDT [2]. Most quantitative experiments are conducted on VisDrone2019, including comparisons with YOLOv8s and other representative object detection models, as well as ablation studies of the proposed components. We also independently train and evaluate both models on UAVDT to examine whether the proposed design remains effective in a different UAV scenario. In addition, representative images from both VisDrone2019 and UAVDT are used for qualitative visualization.

The VisDrone2019 dataset was developed by the AISKYEYE team at the Machine Learning and Data Mining Laboratory of Tianjin University. It is a standard benchmark for UAV vision and fits our setting because small objects account for about 60% to 70% of all instances. The dataset contains 10,209 static images, with 6471 images for training, 548 for validation, and 3190 for testing [29]. It includes about 2.6 million annotated object instances from 10 categories, covering common traffic and urban-scene objects such as pedestrian, people, bicycle, car, van, truck, tricycle, awning-tricycle, bus, and motor. Because of the large number of small objects and the top-down viewing geometry of UAV imagery, VisDrone2019 contains strong scale changes caused by flight altitude and viewing direction, dense object distributions, and frequent occlusion [20]. These factors make detection difficult for standard models and make VisDrone2019 a suitable benchmark for evaluating whether LFC-YOLO can maintain high detection accuracy under strong scale variation and crowded scenes.

The UAVDT dataset was collected from UAV-based urban traffic scenes. It contains images captured under different flight altitudes, camera viewpoints, illumination conditions, weather conditions, and object densities. Compared with VisDrone2019, UAVDT focuses more on vehicle detection and includes many challenging cases, such as small vehicles, background interference, shadows, different object orientations, and partial occlusion. We therefore use UAVDT as a second UAV benchmark, with both models independently trained and evaluated under the same within-dataset protocol.

### 3.3. Evaluation Metrics

We evaluate LFC-YOLO using the standard detection protocol [30]. The main metrics are Precision (*P*), Recall (*R*), and Mean Average Precision (mAP).

Precision measures the proportion of correct positive predictions, while Recall measures the proportion of ground-truth objects that are correctly detected. They are defined as

(3) $$P=\frac{\mathrm{TP}}{\mathrm{TP}+\mathrm{FP}}$$

and

(4) $$R=\frac{\mathrm{TP}}{\mathrm{TP}+\mathrm{FN}}$$

where TP, FP, and FN denote the numbers of true positives, false positives, and false negatives, respectively. These values are computed according to the Intersection over Union (IoU) between predicted bounding boxes and ground-truth boxes.

Mean Average Precision (mAP) is the mean of Average Precision (AP) over all *N* object categories. Each AP is computed from the area under the Precision–Recall curve:

(5) $$\mathrm{mAP}=\frac{1}{N}\sum_{i=1}^{N}{\mathrm{AP}}_{i}$$

We report ${\mathrm{mAP}}_{50}$, which uses an IoU threshold of 0.5, and ${\mathrm{mAP}}_{50:95}$, which averages the results over IoU thresholds from 0.5 to 0.95 with a step size of 0.05. These metrics measure detection performance under different localization criteria. Unless stated otherwise, our own experiments, including the baseline comparison and ablation studies, are evaluated on the VisDrone2019 validation set, which contains 548 images. For comparisons with recently published methods, we additionally report the results available from their original papers as a literature-level reference.

### 3.4. Performance Validation Experiments

We trained the baseline YOLOv8s and the proposed LFC-YOLO separately on the VisDrone2019 dataset to assess the effect of the model changes. Table 2 reports the accuracy, model complexity, and measured speed results.

LFC-YOLO improves ${\mathrm{mAP}}_{50}$ by 4.0 percentage points and ${\mathrm{mAP}}_{50:95}$ by 2.8 percentage points while reducing the parameter count from 11.1 M to 2.9 M. Its nominal operation count also decreases slightly, from 28.5 to 28.0 GFLOPs. However, the measured end-to-end latency increases from 15.7 ms to 19.4 ms, corresponding to a throughput decrease from 63.9 to 51.7 FPS. The accuracy improvement and substantial parameter reduction therefore come with a practical latency cost. These results also show that parameter count, nominal GFLOPs, and measured inference speed describe different aspects of model efficiency and should be reported separately.

**Figure 6:**
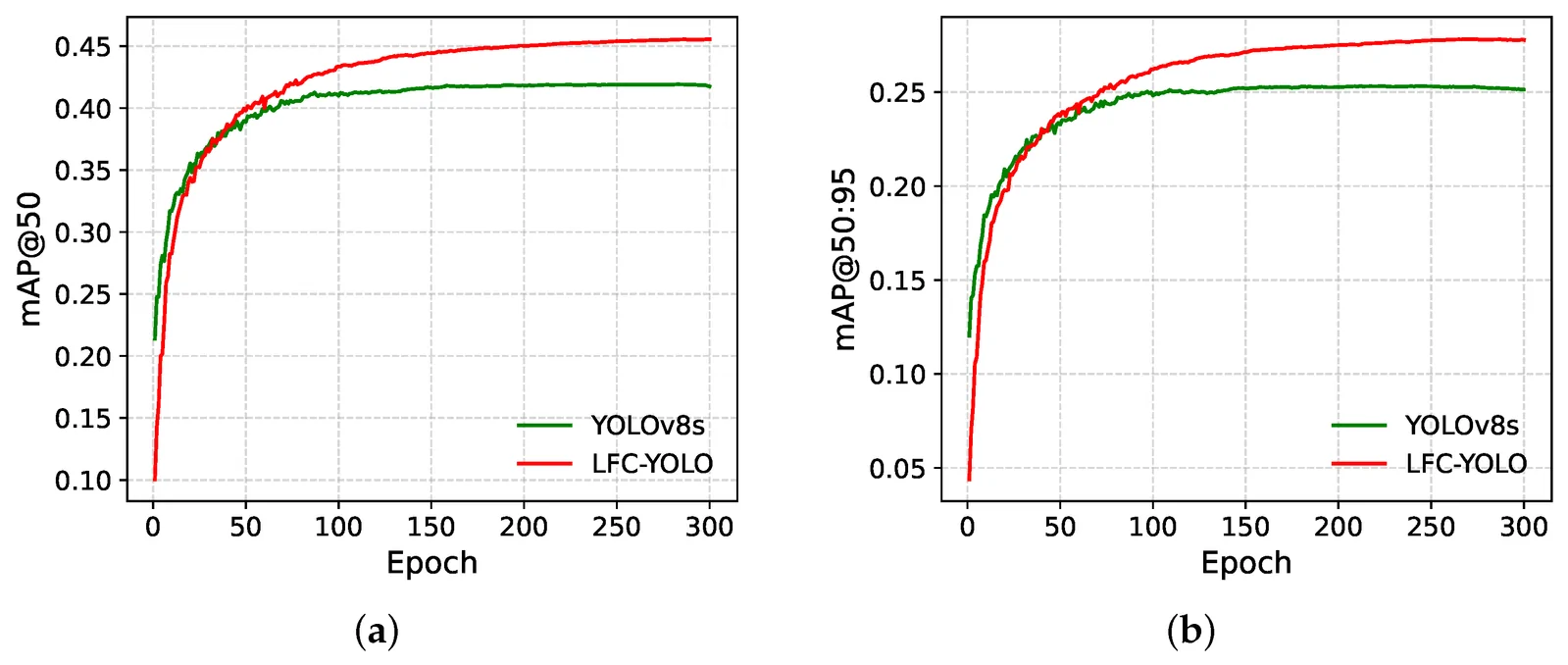
Performance comparison between YOLOv8s and LFC-YOLO on (**a**) ${\mathrm{mAP}}_{50}$ and (**b**) ${\mathrm{mAP}}_{50:95}$.

Figure 6 shows that both models converge under the same training settings and that LFC-YOLO reaches higher final ${\mathrm{mAP}}_{50}$ and ${\mathrm{mAP}}_{50:95}$ values while using substantially fewer parameters.

### 3.5. Ablation Experiments

We conduct ablation experiments on VisDrone2019 to examine how each proposed module affects LFC-YOLO. Starting from the YOLOv8s baseline, we add TSD-Arch, C2f-FCM, and GSConv [12] in different combinations. TSD-Arch changes the feature pyramid to improve tiny-object localization and reduce parameter count. C2f-FCM complements semantic and spatial information in the backbone with a compact decoupled parameterization. GSConv is used in the reconstructed neck to control the extra convolutional cost associated with high-resolution feature maps. Table 3 reports the full ablation results.

TSD-Arch gives the largest single-module accuracy gain. In Model 2, it reduces the parameter count by 70.3% and improves ${\mathrm{mAP}}_{50}$ by 3.5 percentage points over YOLOv8s. Although the shallow high-resolution feature maps increase the computational complexity to 29.8 GFLOPs, Model 2 achieves the lowest latency of 13.9 ms and the highest throughput of 71.9 FPS. This shows that higher GFLOPs do not necessarily result in a longer runtime.

Models 3 and 6, both of which include GSConv, achieve 67.0 and 69.4 FPS, respectively. Model 6 also maintains an ${\mathrm{mAP}}_{50}$ of 45.0%. In contrast, the variants containing C2f-FCM have higher latency than the corresponding variants without C2f-FCM under the current test settings. These results show that GFLOPs alone cannot represent actual inference speed. Further profiling is needed to determine the specific causes of the latency differences.

The complete LFC-YOLO model achieves the highest ${\mathrm{mAP}}_{50}$ and ${\mathrm{mAP}}_{50:95}$ among all evaluated variants. With all three modules, these metrics reach 45.7% and 27.9%, respectively, while the parameter count is reduced to 2.9 M. Its throughput is 51.7 FPS, lower than those of YOLOv8s, Model 2, and Model 6. Although this configuration has higher latency, it is retained because it achieves the best detection accuracy with the smallest parameter count.

### 3.6. Comparative Experiments

We compare LFC-YOLO with two groups of models on the VisDrone2019 dataset. The first group includes representative general YOLO detectors, while the second group consists of methods developed for UAV or small-object detection. Table 4 and Table 5 summarize the results reported in the original publications. Because these studies use different hardware, software, batch sizes, numerical precision, and timing protocols, latency and FPS are not included in the cross-paper comparison.

**Figure 7:**
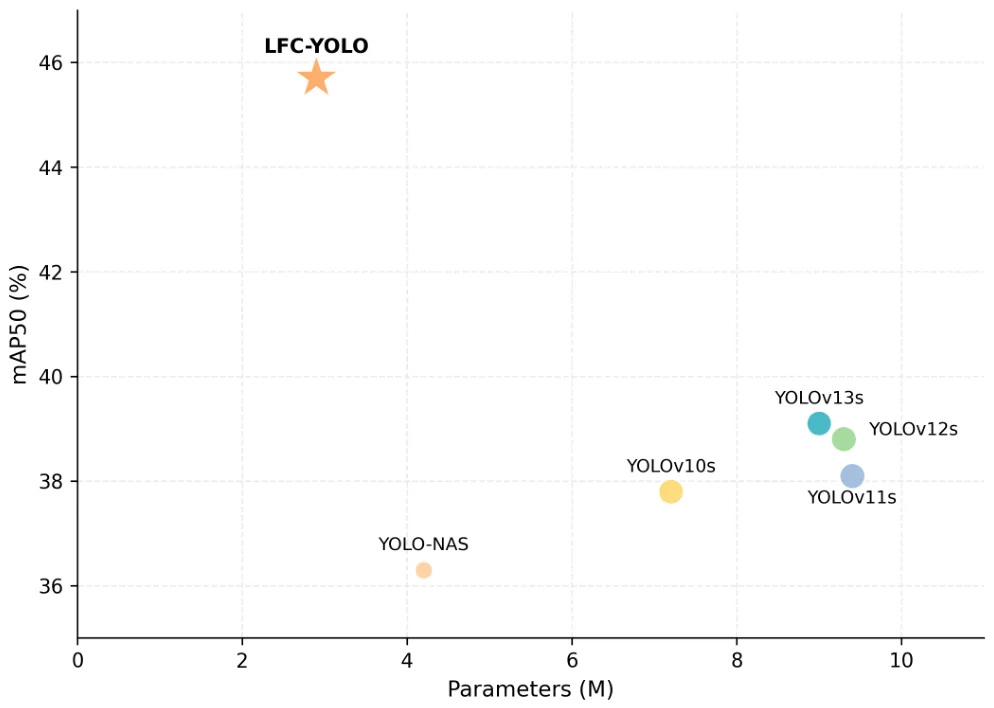
Comparison of the reported ${\mathrm{mAP}}_{50}$ and parameter count of representative general YOLO detectors.

**Figure 8:**
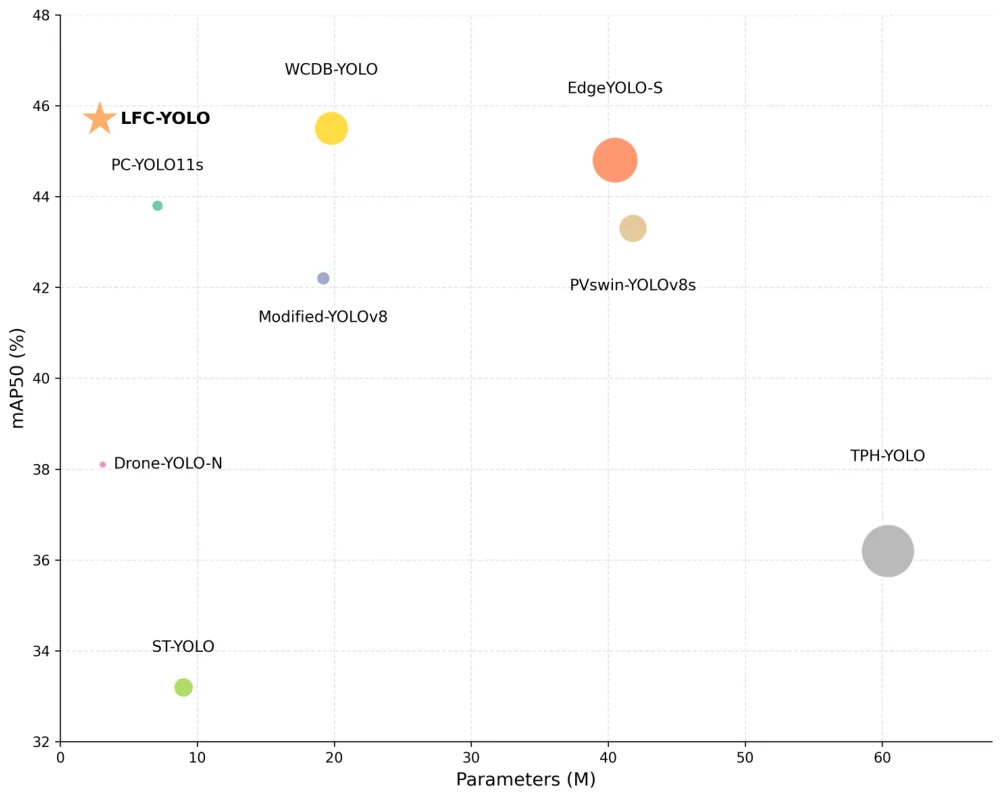
Comparison of the reported ${\mathrm{mAP}}_{50}$ and parameter count of representative UAV and small-object detectors.

Figure 7 and Figure 8 provide a visual summary of the results reported in Table 4 and Table 5. Since the values come from different publications, the figures are used only to compare the reported detection accuracy and model complexity.

Among the methods listed in Table 4, LFC-YOLO has the highest reported ${\mathrm{mAP}}_{50}$ and the smallest parameter count. It exceeds the reported result of YOLOv13s by 6.6 percentage points while using only 2.9 M parameters. However, its 28.0 GFLOPs are higher than those of several general YOLO models. Therefore, the comparison mainly shows that LFC-YOLO obtains higher reported accuracy with a smaller parameter count, rather than lower computational complexity in every respect.

Table 5 shows a similar pattern among UAV and small-object detection methods. Drone-YOLO-N has a comparable parameter count of 3.1 M but reports an ${\mathrm{mAP}}_{50}$ of 38.1%. EdgeYOLO-S reports an ${\mathrm{mAP}}_{50}$ of 44.8% with 40.5 M parameters and 109.1 GFLOPs, whereas WCDB-YOLO reports 45.5% with 19.8 M parameters and 60.1 GFLOPs. LFC-YOLO reports an ${\mathrm{mAP}}_{50}$ of 45.7% with 2.9 M parameters and 28.0 GFLOPs. Since the compared results were obtained under different experimental settings, they should be interpreted only in terms of the reported accuracy, parameter count, and GFLOPs. Based on these values, LFC-YOLO achieves competitive detection accuracy and has the smallest parameter count among the listed methods.

### 3.7. Additional Evaluation on UAVDT

We further train and evaluate LFC-YOLO on the UAVDT dataset [2] to test whether the architectural improvements remain effective in a different UAV scenario. UAVDT is designed for vehicle detection and tracking from UAV videos. It contains about 80,000 representative frames and roughly 0.84 million annotated vehicle instances. The dataset includes dense traffic scenes, complex road environments, and large changes in flight altitude. Both models are trained and evaluated independently on UAVDT.

**Figure 9:**
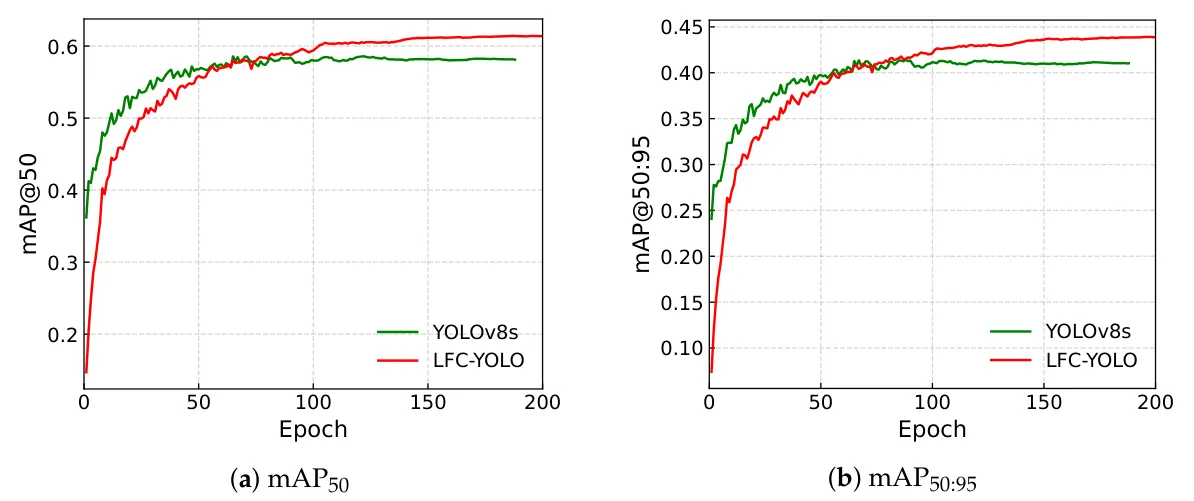
Performance comparison between YOLOv8s and LFC-YOLO on the UAVDT dataset in terms of (**a**) mAP_50_ and (**b**) mAP_50:95_. The curves are shown up to the final epoch reached under the early-stopping strategy.

For a fair comparison, YOLOv8s and LFC-YOLO were trained and evaluated on UAVDT under the same protocol, including the same input size, optimizer settings, maximum epoch budget, and early-stopping strategy. Table 6 reports the quantitative results, and Figure 9 shows the training curves up to the epoch at which training stopped. Compared with YOLOv8s, LFC-YOLO increases ${\mathrm{mAP}}_{50}$ by 3.2 percentage points, ${\mathrm{mAP}}_{50:95}$ by 2.6 percentage points, and precision and recall by 1.3 and 2.9 percentage points, respectively. These results show that the same architectural design remains effective after independent training on UAVDT scenes with dense traffic, vehicle occlusion, and large-scale variation.

As shown in Figure 9, YOLOv8s improves more rapidly during the early training stage, whereas LFC-YOLO progressively catches up and achieves higher final ${\mathrm{mAP}}_{50}$ and ${\mathrm{mAP}}_{50:95}$ values. This observation shows that faster initial convergence does not necessarily translate into better final detection performance. More importantly, LFC-YOLO achieves these improvements while reducing the parameter count from 11.1 M to 2.9 M, indicating that the streamlined network retains the effective feature representation required for the UAVDT detection task. Compared with Figure 6, this trend is more evident on UAVDT.

To further examine how each module affects the results on UAVDT, we conduct an additional ablation study using the same module combinations as those used on VisDrone2019. Table 7 reports the results. The overall trend is consistent with the results on VisDrone2019. TSD-Arch brings the clearest single-module improvement. In Model 2, ${\mathrm{mAP}}_{50}$ increases from 58.3% to 60.3%, and ${\mathrm{mAP}}_{50:95}$ increases from 41.4% to 42.8%. At the same time, the number of parameters decreases from 11.1 M to 3.3 M. The Model 2 results also show that the shallow high-resolution detection design is effective for vehicle detection in UAVDT.

C2f-FCM also improves the baseline when used alone. Model 1 increases ${\mathrm{mAP}}_{50}$ by 1.0 percentage point and ${\mathrm{mAP}}_{50:95}$ by 0.5 percentage points while using fewer parameters and lower GFLOPs than YOLOv8s. GSConv gives a smaller gain when used alone, but it helps reduce redundant computation in the lightweight neck. When GSConv is combined with TSD-Arch, Model 6 reaches the highest ${\mathrm{mAP}}_{50}$ among all variants, with a value of 61.9%.

The complete LFC-YOLO model gives the best overall result on UAVDT. Although it does not achieve the highest value on every metric, it obtains the best ${\mathrm{mAP}}_{50:95}$ of 44.0% and the lowest parameter count of 2.9 M. Compared with YOLOv8s, the full model improves ${\mathrm{mAP}}_{50}$ and ${\mathrm{mAP}}_{50:95}$ while reducing the number of parameters by 73.9%. The accuracy gains observed on both VisDrone2019 and UAVDT show that LFC-YOLO achieves higher mAP values with a substantially lower parameter count.

### 3.8. Visualization Results

**Figure 10:**
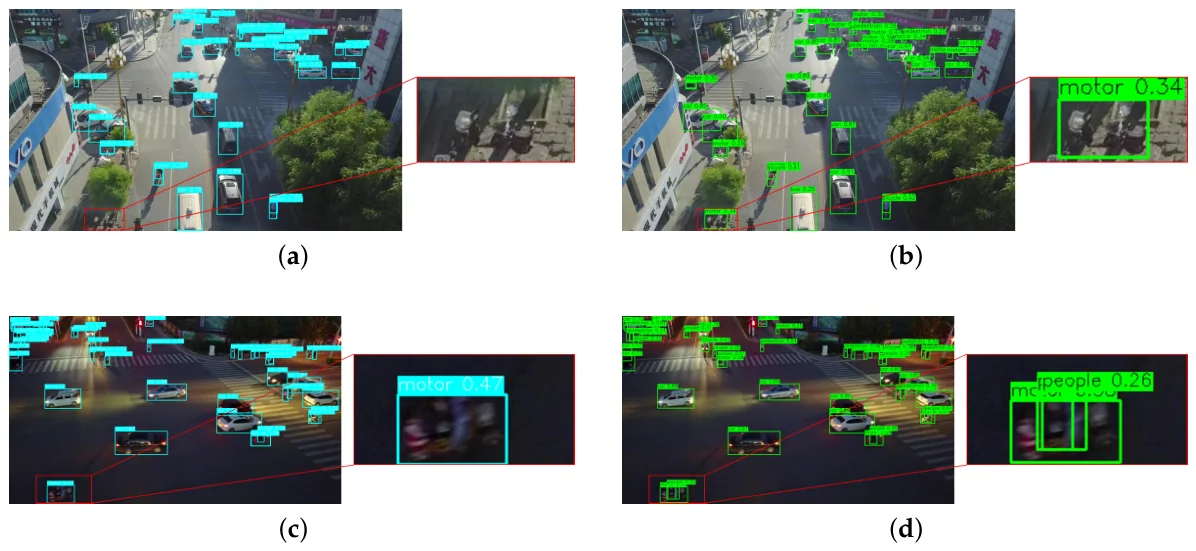
Detection results on the VisDrone2019 dataset. (**a**,**c**) show the outputs of YOLOv8s, and (**b**,**d**) show the outputs of LFC-YOLO.

We use representative aerial images from the VisDrone2019 dataset to compare LFC-YOLO with the YOLOv8s baseline. The visual results are shown in Figure 10.

In Figure 10a, YOLOv8s misses a motorcycle that is occluded in the lower-left region. In Figure 10c, it also fails to detect a heavily occluded pedestrian. LFC-YOLO detects these objects in both cases, as shown in Figure 10b,d. These examples suggest that LFC-YOLO is less affected by occlusion and cluttered backgrounds and that it reduces missed detections for small objects in dense aerial scenes.

**Figure 11:**
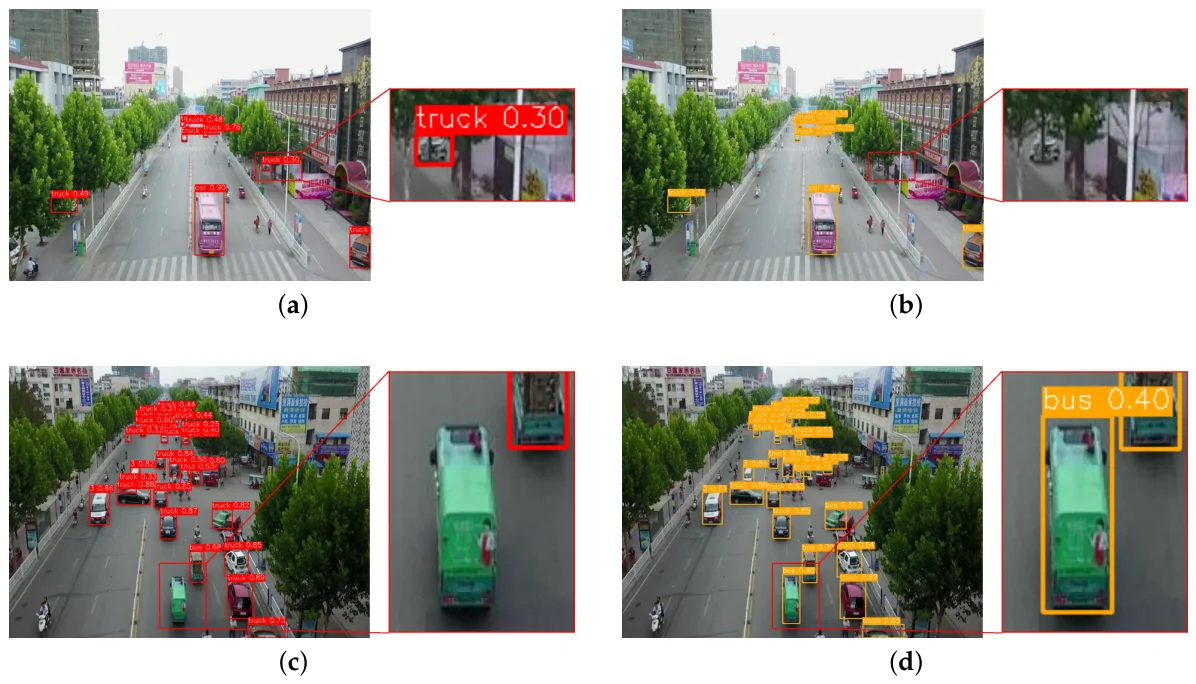
Detection results on the UAVDT dataset. (**a**,**c**) show the outputs of YOLOv8s, and (**b**,**d**) show the outputs of LFC-YOLO.

We also compare the two models on representative samples from the UAVDT dataset to examine their behavior in another UAV scenario. As shown in Figure 11a, YOLOv8s misclassifies a vehicle under a tree as a truck, while LFC-YOLO avoids this error. In Figure 11c, YOLOv8s misses the bus near the bottom center of the image, whereas LFC-YOLO detects the target. These examples illustrate cases in which LFC-YOLO avoids a missed detection or category confusion produced by the baseline in complex UAVDT scenes.

## 4. Discussion

The experimental results show that the improvement of LFC-YOLO mainly comes from adapting the feature hierarchy to the scale distribution of UAV objects. In VisDrone2019, many objects occupy only a small number of pixels, and repeated downsampling can easily weaken their spatial cues. By removing the deepest $P_{5}$ branch and introducing a shallow $\left\{P_{2},P_{3},P_{4}\right\}$ feature pyramid, TSD-Arch allows the detector to operate on higher-resolution feature maps. This interpretation is supported by the ablation results, where TSD-Arch provides the largest single-module accuracy gain in the ablation study. However, the use of high-resolution features also increases the size of intermediate activations and may increase memory-access and feature-fusion overhead, which motivates the use of lightweight operations in the reconstructed neck.

The roles of C2f-FCM and GSConv are different from that of TSD-Arch. C2f-FCM improves feature representation in the backbone by complementing semantic and spatial information, helping the model distinguish small targets from cluttered UAV backgrounds. GSConv, by contrast, is used to control the convolutional cost of the reconstructed high-resolution neck. The ablation results support this interpretation: GSConv alone brings only a limited accuracy gain, but it helps maintain a compact model when combined with the shallow feature pyramid. The final improvement therefore results from the combination of scale-aware feature reconstruction, semantic–spatial feature complementation, and lightweight neck computation.

The substantial reduction in parameter count is another important result of this work. Statistical learning theory emphasizes that model capacity should be considered in relation to the amount and diversity of labeled data [13,14]. Parameter count is not equivalent to the VC dimension, and fewer parameters do not by themselves guarantee better generalization. Nevertheless, theoretical bounds for piecewise-linear networks depend on both the number of weights and network depth [43], making it meaningful to examine whether task performance can be preserved with less capacity. In our experiments, LFC-YOLO reduces the parameter count from 11.1 M to 2.9 M while achieving higher detection accuracy. This suggests that, after substantial simplification of the deep stages, the model still preserves the task-relevant feature representations needed for the evaluated detection tasks. This result should not be interpreted as evidence that reducing the parameter count always improves generalization.

The UAVDT results provide further evidence that the proposed design remains effective after independent training on a second UAV dataset. Model 6 obtains the highest ${\mathrm{mAP}}_{50}$, whereas the complete LFC-YOLO model achieves the highest ${\mathrm{mAP}}_{50:95}$ and the lowest parameter count. Their ${\mathrm{mAP}}_{50}$ values differ by only 0.4 percentage points. The higher ${\mathrm{mAP}}_{50:95}$ of the complete model indicates stronger aggregate performance across IoU thresholds from 0.50 to 0.95. Together with the training curves in Figure 9, these results suggest that the streamlined model retains the feature representation required for vehicle detection on UAVDT.

The fixed 640×640 input resolution remains a limitation of the present evaluation. Although letterbox preprocessing preserves the original aspect ratio, downscaling native high-resolution UAV imagery may compress very small targets to only a few pixels, weakening or removing texture and boundary cues before feature extraction. LFC-YOLO reduces the subsequent information loss caused by network downsampling through its shallow $\left\{P_{2},P_{3},P_{4}\right\}$ feature pyramid, but it cannot recover information that has already been discarded during input preprocessing. The reported results therefore describe performance under the standard 640×640 benchmark setting rather than under native-resolution deployment. Higher-resolution inputs, image tiling, and adaptive-resolution processing should be further studied together with their effects on detection accuracy and latency.

Finally, the measured timing results require a more precise interpretation of the practical efficiency of LFC-YOLO. Under the same end-to-end detection-pipeline timing protocol, YOLOv8s requires 15.7 ms per image, corresponding to 63.9 FPS, whereas LFC-YOLO requires 19.4 ms per image, corresponding to 51.7 FPS. Thus, although LFC-YOLO reduces the parameter count by 73.9% and slightly lowers GFLOPs, these reductions do not translate into lower latency on the tested GPU. This result shows that parameter count and nominal operation count describe only part of practical inference efficiency. The shallow $\left\{P_{2},P_{3},P_{4}\right\}$ feature pyramid processes higher-resolution feature maps and may introduce additional intermediate-activation, memory-access, and feature-fusion overhead, although identifying the exact source of the latency difference would require operator-level profiling. LFC-YOLO therefore provides its main advantage by substantially reducing the parameter count while improving detection accuracy, although its per-image latency is higher than that of YOLOv8s under the current timing protocol. These results reflect a trade-off among detection accuracy, model size, and practical inference speed. Since the timing evaluation was conducted only on an NVIDIA GeForce RTX 4090, the latency, throughput, and deployment feasibility of LFC-YOLO on onboard UAV computing platforms require further evaluation.

## 5. Conclusions

This paper presents LFC-YOLO, a parameter-efficient detector for small objects in UAV imagery. It combines a shallow high-resolution feature pyramid with semantic–spatial feature complementation and lightweight neck operations. TSD-Arch removes the conventional $P_{5}$ branch to preserve spatial information for small targets, C2f-FCM complements spatial and semantic features, and GSConv controls the convolutional cost of the reconstructed neck. On VisDrone2019, LFC-YOLO reduces the parameter count from 11.1 M to 2.9 M while improving ${\mathrm{mAP}}_{50}$ by 4.0 percentage points. Under the unified end-to-end detection-pipeline timing protocol, LFC-YOLO requires 19.4 ms per image and achieves 51.7 FPS on an NVIDIA GeForce RTX 4090, whereas YOLOv8s requires 15.7 ms per image and achieves 63.9 FPS. The results therefore reveal a clear trade-off among detection accuracy, parameter count, and measured detection-pipeline speed. Independent training and evaluation on UAVDT further show that the proposed architecture remains effective on a second UAV benchmark.

This study is limited by the fixed 640×640 input size and the absence of measurements on representative onboard processors. Future work will investigate higher-resolution and image-tiling strategies, evaluate LFC-YOLO on UAV onboard computing platforms, and improve its robustness under low-light and adverse-weather conditions.

## Figures and Tables

**Table 1:** Hyperparameter settings for model training.

Hyperparameter	Value
Input Size	640×640
Maximum Epochs	300
Patience	100
Batch Size	8
Optimizer	SGD
Initial Learning Rate	0.01
Momentum	0.937
Weight Decay	0.0005
Data Augmentation	Mosaic
Number of Workers	8

**Table 2:** Comparison of YOLOv8s and LFC-YOLO in accuracy, model complexity, and end-to-end detection-pipeline speed.

Models	*P* (%)	*R* (%)	mAP_50_ (%)	mAP_50:95_ (%)	Params (M)	GFLOPs	Latency (ms)	FPS
YOLOv8s	51.4	41.0	41.7	25.1	11.1	28.5	15.7	63.9
LFC-YOLO	55.7	43.4	45.7	27.9	2.9	28.0	19.4	51.7

**Table 3:** Ablation study results of the proposed modules on the VisDrone2019 dataset, including model complexity and end-to-end detection-pipeline speed.

Models	C2f-FCM	TSD-Arch	GSConv	*P* (%)	*R* (%)	mAP_50_ (%)	mAP_50:95_ (%)	Params (M)	GFLOPs	Latency (ms)	FPS
YOLOv8s				51.4	41.0	41.7	25.1	11.1	28.5	15.7	63.9
1	✓			52.7	40.6	42.4	25.5	10.5	27.5	19.6	51.0
2		✓		55.9	42.8	45.2	27.4	3.3	29.8	13.9	71.9
3			✓	51.3	41.5	41.7	25.3	10.7	28.0	14.9	67.0
4	✓		✓	53.4	39.9	41.9	25.3	10.2	27.0	17.6	56.8
5	✓	✓		55.8	42.5	45.2	27.4	3.1	28.4	18.9	52.8
6		✓	✓	55.3	43.0	45.0	27.5	3.2	29.6	14.4	69.4
LFC-YOLO	✓	✓	✓	55.7	43.4	45.7	27.9	2.9	28.0	19.4	51.7

Note: ✓ indicates that the corresponding module is included in the model.

**Table 4:** Comparison of reported results for general YOLO detectors on the VisDrone2019 dataset.

Model	mAP_50_ (%)	Params (M)	GFLOPs
YOLO-NAS [31]	36.3	4.2	10.6
YOLOv10s [32]	37.8	7.2	20.9
YOLOv11s [33]	38.1	9.4	21.6
YOLOv12s [34]	38.8	9.3	21.4
YOLOv13s [35]	39.1	9.0	20.8
LFC-YOLO	45.7	2.9	28.0

Note: Results for the comparison methods are taken from their original publications, whereas LFC-YOLO is evaluated using our implementation with an input size of 640×640. Runtime results are omitted because the studies use different hardware and timing protocols.

**Table 5:** Comparison of reported results for UAV and small-object detection models on the VisDrone2019 dataset.

Model	mAP_50_ (%)	Params (M)	GFLOPs
PC-YOLO11s [36]	43.8	7.1	–
EdgeYOLO-S [37]	44.8	40.5	109.1
Drone-YOLO-N [38]	38.1	3.1	–
ST-YOLO [39]	33.2	9.0	20.1
WCDB-YOLO [40]	45.5	19.8	60.1
Modified-YOLOv8 [41]	42.2	19.2	9.7
PVswin-YOLOv8s [42]	43.3	41.8	–
TPH-YOLO [20]	36.2	60.4	145.7
LFC-YOLO	45.7	2.9	28.0

Note: Results for the comparison methods are taken from their original publications, whereas LFC-YOLO is evaluated using our implementation with an input size of 640×640. Runtime results are omitted because the studies use different hardware and timing protocols.

**Table 6:** Performance comparison on UAVDT under the same training and evaluation settings.

Model	*P* (%)	*R* (%)	mAP_50_ (%)	mAP_50:95_ (%)
YOLOv8s	69.0	53.2	58.3	41.4
LFC-YOLO	70.3	56.1	61.5	44.0

**Table 7:** Ablation study results of the proposed modules on the UAVDT dataset.

Models	C2f-FCM	TSD-Arch	GSConv	*P* (%)	*R* (%)	mAP_50_ (%)	mAP_50:95_ (%)	Params (M)	GFLOPs
YOLOv8s				69.0	53.2	58.3	41.4	11.1	28.4
1	✓			70.7	54.0	59.3	41.9	10.6	27.5
2		✓		69.0	55.2	60.3	42.8	3.3	29.8
3			✓	71.1	53.7	59.1	41.7	10.8	28.0
4	✓		✓	70.0	54.5	59.6	41.6	10.2	27.0
5	✓	✓		70.1	56.3	61.6	43.5	3.1	28.4
6		✓	✓	70.7	56.1	61.9	43.4	3.2	29.3
LFC-YOLO	✓	✓	✓	70.3	56.1	61.5	44.0	2.9	27.9

Note: ✓ indicates that the corresponding module is included in the model.

## Data Availability

Publicly available datasets were analyzed in this study. The VisDrone2019 dataset is available at https://github.com/VisDrone/VisDrone-Dataset (accessed on 15 March 2026), and the UAVDT dataset is available at https://sites.google.com/view/grli-uavdt/ (accessed on 15 March 2026). The experimental results supporting the findings of this study are included in the article. The implementation code is available from the corresponding author upon reasonable request.
